# Case Report: Disseminated herpes simplex virus complicated by hemophagocytic lymphohistiocytosis in a neonate

**DOI:** 10.3389/fped.2025.1571715

**Published:** 2025-07-18

**Authors:** Nicholas Tadros, Nihal Godiwala

**Affiliations:** Department of Pediatrics, LSU Health Sciences Center New Orleans, New Orleans, LA, United States

**Keywords:** neonatal herpes simplex, hemophagocytic lymphohistiocytosis, disseminated infection, intensive care, immunosuppression

## Abstract

Neonatal herpes simplex virus (HSV) infection carries a high mortality rate due to its potential to cause disseminated disease involving multiple organ systems, which can rapidly progress to shock and death if not promptly treated. In rare cases, neonates may mount an uncontrolled inflammatory response leading to hemophagocytic lymphohistiocytosis (HLH), a severe hyperinflammatory syndrome. We present a case of neonatal HSV infection complicated by HLH, highlighting the challenges of managing both conditions concurrently. Our therapeutic approach demonstrated a reduction in systemic inflammation and viral load; however, despite these efforts, the patient developed multiorgan failure and ultimately died from the initial disease process. This case underscores the severity of neonatal HSV infection and emphasizes the critical role pediatricians play in early identification of transmission risk factors and prevention strategies.

## Introduction

Early-onset neonatal sepsis, defined as sepsis occurring within the first 72 h of life, carries a high mortality rate of up to 54% (1). A substantial body of research has focused on identifying its causes and risk factors, as well as improving management strategies. Among the most lethal etiologies is herpes simplex virus (HSV) infection, which can be transmitted vertically from the mother or postnatally through contact with an active herpetic lesion (2–4). The clinical presentation of neonatal HSV infection ranges from localized involvement of the skin, eyes, and mouth—with near-zero mortality—to disseminated disease, which has a reported mortality rate of approximately 29% (2–5). In rare cases, neonatal HSV infection may be further complicated by hemophagocytic lymphohistiocytosis (HLH).

HLH is a life-threatening hyperinflammatory syndrome triggered by excessive immune activation, with a mortality rate as high as 42% (6, 7). Primary HLH is typically associated with genetic mutations, whereas secondary HLH may be triggered by infections, malignancies, or rheumatologic conditions (6). Infections most commonly associated with HLH include Epstein–Barr virus (EBV), cytomegalovirus (CMV), and HSV, with EBV being the most frequent culprit (4, 6). Managing HLH in the setting of an active infection poses a significant clinical challenge, as standard HLH treatment involves immunosuppression, which can impair the host's ability to control the underlying infection (4, 6).

Here, we present a case illustrating the complexity of managing disseminated neonatal HSV infection complicated by secondary HLH.

## Case presentation

The patient is a 5-day-old female, born at 38.3 weeks’ gestation, who was transferred from an outside hospital for evaluation of continuous renal replacement therapy (CRRT) in the setting of HSV encephalitis complicated by acute hepatic and renal failure, with concern for hemophagocytic lymphohistiocytosis (HLH).

She was born via cesarean section to a 29-year-old G2P2 mother who received full prenatal care and had negative prenatal infectious screening. Labor was complicated by maternal fever of unknown origin, prompting cesarean delivery for failure to progress. During labor, the mother received cefazolin, azithromycin, and acyclovir. The delivery was uncomplicated, and the patient's Apgar scores were 8 and 9 at 1 and 5 min, respectively. She received routine resuscitation and was initially managed in the newborn nursery. Initial laboratory evaluations, including a blood culture obtained due to maternal fever, were within normal limits; the blood culture remained negative at 48 h. The patient was discharged to room-in with her mother on the third day of life with no concerns.

Two days later, the patient developed cyanosis, lethargy, poor oral intake, and decreased urine output. She was brought to the emergency department, where initial evaluation included complete blood count, comprehensive metabolic panel, lactate level, chest radiograph, and lumbar puncture. Laboratory results were notable for markedly elevated aspartate aminotransferase (AST) and alanine transaminase (ALT). Empiric antibiotic therapy with ampicillin and gentamicin was initiated, and the patient was admitted to the neonatal intensive care unit (NICU) for further management.

In the NICU, acyclovir was added after it was discovered that the mother was being treated in the ICU for HSV encephalitis, confirmed by cerebrospinal fluid (CSF) polymerase chain reaction (PCR). On admission, laboratory results revealed acute hepatitis and hyperferritinemia, with AST and ALT levels >1,000 U/L and ferritin >33,000 µg/L. Pediatric gastroenterology recommended additional workup, including ammonia, gamma-glutamyl transferase (GGT), international normalized ratio (INR), and partial thromboplastin time (PTT), all of which were grossly abnormal. The patient was treated with lactulose for hyperammonemia and received methylprednisolone bursts for suspected HLH. Fresh frozen plasma (FFP), vitamin K, and cryoprecipitate were administered to address coagulopathy.

Brain MRI was unremarkable; however, video EEG revealed diffuse cerebral dysfunction. CSF studies later confirmed HSV-1 encephalitis. The patient's condition continued to deteriorate, with progression to anuric renal failure, prompting transfer to our center for CRRT and further management of multiorgan failure due to disseminated HSV with suspected HLH.

On admission, physical examination revealed a sedated, mechanically ventilated infant with icterus, tachycardia, diminished breath sounds, delayed capillary refill, petechiae, and hepatosplenomegaly. Laboratory evaluation confirmed anuric renal failure, acute hepatic failure, and disseminated HSV-1 infection. Pediatric hematology was consulted and recommended further testing for HLH markers, including soluble IL-2 receptor (sCD25), CD107a-positive natural killer (NK) cell activity, mean channel fluorescence (MCF), and CXCL9.

The results were as follows:

• **sCD25**: 4,646 U/ml (reference: 398–1,490 U/ml)
• **CD107a POS NK**: 29% (reference: 11%–35%)
• **CD107a MCF**: 388 MCF (reference: 207–678)
• **CXCL9**: 5,388 pg/ml (reference: <647 pg/ml)

Based on these findings and the clinical picture, the patient was diagnosed with disseminated HSV-1 infection complicated by secondary HLH, along with acute hepatic and anuric renal failure.

## Discussion

Herpes simplex virus (HSV) is highly prevalent in the United States (8). Although typically mild in adolescents and adults, neonatal HSV infection can have devastating consequences, underscoring the importance of early recognition by outpatient providers and nursery hospitalists (4). There are two types of HSV: HSV-1, commonly associated with oral lesions, and HSV-2, typically linked to genital lesions (2, 3). Both types may present with vesicular skin eruptions and generalized malaise (2–4). HSV-2 is the most common cause of vertical transmission (3, 4).

Asymptomatic pregnant women are not routinely screened for HSV in the outpatient setting. However, if active genital lesions are observed on physical exam, cesarean delivery is recommended to reduce the risk of transmission (9). Neonates are at greatest risk when maternal infection occurs around the time of delivery, with transmission rates reaching up to 60% (4). In our case, limited maternal history made the route of transmission unclear. However, given the infant's positive HSV-1 PCR, we infer an oral route of transmission, possibly from contact with an unrecognized herpetic lesion.

**Figure 1:**
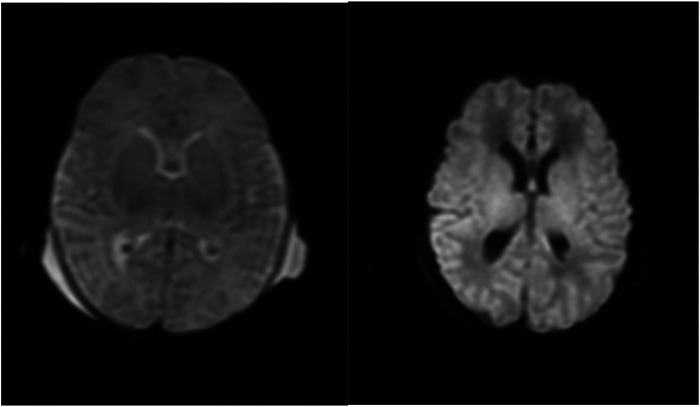
MRI brain with contrast from 7/19. Diffusion-weighted imaging depicting restricted diffusion predominantly involving the bilateral ventrolateral thalami and adjacent putamina with possible cortical restricted diffusion predominantly involving the peri-Rolandic cortices consistent with global cerebral injury.

Neonatal HSV infection can present in one of three ways. The most common manifestation is limited cutaneous involvement (4). Central nervous system (CNS) involvement is the second most frequent presentation, typically manifesting as encephalitis with altered mental status and temporal lobe findings on imaging (9). The most severe form, disseminated disease, occurs in approximately 25% of cases and results in multiorgan involvement, including the lungs, liver, and kidneys (4). Our patient had disseminated HSV as evidenced by fulminant hepatic failure, acute respiratory distress syndrome, and hypoxic–ischemic encephalopathy (Figure 1). Unfortunately, her clinical course was further complicated by HLH.

HLH is a hyperinflammatory syndrome caused by an excessive immune response to infectious, malignant, or autoimmune triggers (6). Although rare in neonates, the risk of HLH increases significantly in the setting of HSV infection (6, 7). HLH should be suspected in cases of persistent inflammation despite appropriate management of the primary infection and can be assessed using the HScore (10). Diagnosis is confirmed by evaluating immunologic markers such as soluble IL-2 receptor (sCD25), IL-18, CD163, and CXCL9 (6).

In our patient, the HScore was calculated at 244, which correlates with a >99% probability of HLH (10). The calculation was based on the following parameters: temperature of 103°F (33 points), hepatosplenomegaly (38 points), hemoglobin of 7.4 g/dl and platelet count of 54 × 10^3^/µl (24 points), ferritin of 33,511 ng/ml (50 points), triglycerides of 523 mg/dl (64 points), AST of 476 U/L, and absence of bone marrow biopsy (0 points) (10).

To evaluate for primary HLH, the *Invitae Hereditary HLH Disorders Panel* (GTR000553816.2) was sent (11). This panel screens for genetic mutations associated with familial HLH and aids in diagnosis, risk assessment, and therapeutic planning. The results were negative, further supporting a diagnosis of secondary HLH. Additional confirmatory tests, including sCD25, CD107a, and CXCL9, also supported the diagnosis (Table 1). The only limitation in diagnostic workup was the inability to obtain a bone marrow biopsy due to the patient's clinical instability.

**Table 1:** HLH-specific markers. This table shows trends in HLH-specific markers before and after treatment with steroids and etoposide. The treatment plan for HLH started on Day 2.

Day	1	2	3	4	5	6	7	8	9	10	11	12	13	14	15	16
Triglycerides (mg/dl)	480			525						108		248				
Ferritin (ng/ml)	40,000	40,000	40,000	40,000	40,000	40,000	40,000	40,000	40,000	40,000	26,374	17,972	11,886	9,848	5,910	4,699
IL-2 (U/ml)					4,646					3,030						3,532
CXCL-9 (pg/ml)			5,388								3,740					

Management was multifaceted. Acyclovir was initiated at 20 mg/kg every 8 h to treat HSV, and viral load monitoring confirmed response. HLH treatment requires immunosuppression, which can exacerbate active infection (12). In this case, the benefits of treatment outweighed the risks, and the patient was started on methylprednisolone at 10 mg/m^2^ for 14 days, followed by a taper to 5 mg/m^2^ for an additional 14 days. Toward the end of the steroid course, the HLH-94 protocol was initiated, and the patient received etoposide at a reduced dose of 75 mg/m^2^ (50% of standard dosing) due to hepatic dysfunction (13).

HLH biomarkers were trended during treatment and showed an appropriate response (Table 1). Despite this, the patient developed irreversible organ damage, including fulminant hepatic failure, acute respiratory distress syndrome, renal failure, and hypoxic–ischemic encephalopathy (Figure 1). Her condition continued to deteriorate, requiring maximal vasopressor support, daily blood products for severe coagulopathy, and escalating mechanical ventilation due to worsening pulmonary compliance. After careful consideration and multidisciplinary discussions, the patient's family made the decision to transition to hospice care, and the patient peacefully passed away.

## Conclusion

We presented a case of disseminated neonatal HSV infection complicated by HLH that progressed to multisystem organ failure and death. This case illustrates the potentially lethal nature of neonatal HSV and emphasizes the vital role pediatricians and nursery clinicians play in identifying maternal risk factors that predispose to vertical transmission. Early recognition and initiation of antiviral therapy are essential in preventing disease progression and reducing complications associated with this devastating infection.

## Data Availability

The original contributions presented in the study are included in the article/Supplementary Material, further inquiries can be directed to the corresponding author.
